# Integration of complementary and alternative medicine in the Indian health system: how the state inadvertently undermines policy implementation

**DOI:** 10.1093/heapol/czag025

**Published:** 2026-02-27

**Authors:** Gupteswar Patel, Caragh Brosnan, Ann Taylor

**Affiliations:** Community and Health Research Unit, School of Health and Care Sciences, University of Lincoln, LMS3001, Ross Lucas Medical Sciences Building, Brayford Pool Campus, Lincoln LN6 7TS, United Kingdom; School of Humanities, Creative Industries and Social Sciences, The University of Newcastle, University Drive, Callaghan, NSW, 2308, Australia; School of Humanities, Creative Industries and Social Sciences, The University of Newcastle, University Drive, Callaghan, NSW, 2308, Australia; School of Humanities, Creative Industries and Social Sciences, The University of Newcastle, University Drive, Callaghan, NSW, 2308, Australia

**Keywords:** AYUSH integration, health-system governance, biomedicalization, state’s role

## Abstract

India’s AYUSH (Ayurveda, Yoga and Naturopathy, Unani, Siddha, and Homeopathy) integration policy emphasizes medical pluralism. However, implementation occurs within a complex health system where the state apparatus, through its governance and policy processes, affects health services and outcomes. This study explores how state and policy complexities shape AYUSH integration processes and practitioners’ capacities in primary healthcare. Qualitative research was conducted in an eastern Indian state and involved observations (19 days) and interviews (37) with AYUSH doctors, biomedical doctors, nurses, pharmacists, and administrators. Thematic analysis enabled identification of themes. State-level employment rules placed AYUSH doctors on low-paid, short-term rolling contracts, but the effects of this marginalized position were intensified by irregular AYUSH medicine supplies and shared governance between two directorates. Governance of integrative facilities and AYUSH medicine stock-outs shifted practice patterns towards biomedical treatments by AYUSH doctors to keep health services functioning, which increased biomedicine demand and further narrowed the scope of AYUSH in a self-reinforcing cycle. Inter-departmental collaboration between the directorates was fragmented, lacking accountability and prioritization of AYUSH integration activities. Limitations in AYUSH medicines and the absence of promotional campaigns narrowed the scope of AYUSH services and facilitated the ‘biomedicalization’ of AYUSH integration. Local governance bodies offered occasional support, but their involvement was neither formalized nor consistent. Thus, integration processes emerged not from linear policy structures but from feedback mechanisms in which changes in policy priorities at the state and district levels produced disproportionate effects on AYUSH integration, demonstrating a system responsive to resource and information flows. Achieving medical pluralism will require adaptive governance: setting iterative integration targets, establishing cross-directorate collaboration and learning platforms, and increasing the resource independence of AYUSH.

Key messages

- State policy design and day-to-day administrative routines set the boundaries for AYUSH integration, determining how they are interpreted to deliver services.
- Resource bottlenecks, fragmented governance, and bureaucratic frictions within the state apparatus divert implementation from its pluralist aims, narrowing space for AYUSH practice.
- Through funding decisions, staffing rules, weak inter-governmental collaboration, and local governance, the state defines implementation realities, exerting disproportionate influence over the AYUSH integration.
- There is the state's constitutive role in policy implementation and redefining the trajectory of health system reforms like AYUSH integration.

## Introduction

Policy planning, implementation, and evaluation are inherently complex processes influenced by various factors and actors. Evidence-based approaches, stakeholder engagement, and institutional structures shape policy decision-making and policy practices (Barnes et al. 2003). Complexity theory highlights the dynamics of policymaking, where diverse professionals and unforeseeable factors require development of adaptive strategies for effective implementation (Byrne 1998, Haynes 2008, Greenhalgh and Papoutsi 2018). This occurs at multiple levels, facilitated by ‘street-level bureaucrats’ who interpret and adapt policies to local contexts, resulting in variable outcomes (Lipsky 1980). Policy and program evaluation involves assessing the interplay of institutions, interests, ideas, and networks, where networks act as pathways for adopting ideas and balancing power among stakeholders to achieve consensus (Shearer et al. 2016). However, limitations in policy implementation include the unpredictability of network dynamics, and the challenges posed by established institutional norms and power imbalances (Shearer et al. 2016). These limitations highlight the need for flexible and iterative approaches to policies and their implementation, often leading to (un)desirable outcomes.

Thus, unintended corollaries of policy implementations are not uncommon in the public sector, due to interactions between sets of guidelines and the complex systemic contexts in which implementation occurs (Barthwal and Sah 2008), in this case, within the health system. This paper examines how system complexities and governance processes shape the implementation of an integrative health policy in India.

Since 2005, the government of India has supported and implemented a policy of integrating traditional medical systems—Ayurveda, Yoga and Naturopathy, Unani, Siddha, and Homeopathy (collectively referred to as AYUSH in policy and practice)—into the public health system. The government support has been reiterated in the recent Ministry of Health and Family Welfare 2017, and National Education Policy, 2020, calling for stronger medical pluralism in the public health system. However, challenges in the real-world contexts of the public health system have limited the extent of the integration (Patel et al. 2023). While systemic barriers are acknowledged in existing literature, the ways in which the state mediates the AYUSH integration policy process remain inadequately explored. Effective integration requires that the policy actors embrace risks and challenges, and incorporate governmental factors in the implementation processes. Understanding the role of the state in AYUSH integration is important, as state-led resources, leadership, and governance shape both the opportunities and challenges in the implementation. The complexities of the health systems and policy environment, including a range of administrative levels and service types, further illustrate the impact of state decisions.

### Background

India’s public health system is governed collaboratively by central and state governments, with increasing involvement from local governance bodies. At the central level, the Ministry of Health and Family Welfare (MoHFW) formulates national health policies, designs programmes, and provides partial funding and technical guidance. Agencies under MoHFW, including the National Health Mission, set standards and perform regulatory and evaluative roles, devising national priority areas such as AYUSH integration, maternal-child health, and pandemic preparedness.

Health remains primarily a state matter under India's constitution, with states responsible for adapting national policies to local contexts, managing health infrastructure, staffing, and procurement of medical supplies. While states receive partial central funding, they significantly contribute to health system financing and administration. Increasing decentralization is evident through Village Health Sanitation and Nutrition Committees (VHSNC) and Panchayati Raj Institutions, (elected local bodies responsible for overseeing local governance) (Kumar and Mishra 2016).

Globally, there is a growing interest in traditional, complementary, and alternative medicine (TCAM), and these systems of medicine are being integrated into healthcare systems in countries such as Brazil, China, Colombia, Israel and Mexico (World Health Organization 2013). In India, AYUSH are recognized TCAM systems regulated by the Ministry of AYUSH, and the AYUSH systems of medicine are epistemologically distinct from the biomedicine.

Modern biomedicine is anchored in a naturalistic, reductionist understanding of the human body, framing illness as malfunction in identifiable anatomical and physiological structures (organs, tissues, cells and biochemical pathways). Health is defined as numerically measurable physiological normality, and illness is considered a deviation to be measured and classified by objective criteria. This epistemic stance underpins a disease-centred ethic in which diagnostic categories determine medical intervention targeting organs. Despite calls for person-centred and biopsychosocial approaches, its philosophical core remains mechanistic and orientated towards discreate disease control (see Ning 2008).

By contrast, AYUSH systems of medicine depend on distinct metaphysics, therapeutic aims and ontologies of health and illness. For example, Ayurveda conceptualizes health as dynamic equilibrium among the ‘tridosha’*—*three fundamental humours—[‘vata’ (wind), ‘pitta’ (bile), and ‘kapha’ (phlegm)] across physical, psychological, and spiritual domains. Therefore, illness is characterized by ‘doshic’ imbalance and environmental context rather than discrete disease entities alone (Jayasundar 2010, Yadav 2017). Similarly, Unani medicine defines illness as an imbalance between the four humours (blood, phlegm, yellow bile, and black bile) within an elemental principle, with Siddha placing an emphasis on rejuvenation and spiritual realization (Subbarayappa 1997, Syeda et al. 2004). These systems adopt a vitalistic, harmony-focused approach that contrasts with biomedicine’s reductionist, organ-centred focus.

In 2005, the Indian government, seeking to address a persistent shortage of biomedical doctors, began integrating AYUSH professionals into primary healthcare (Samal 2015). In 2020, India had one biomedical doctor per 1428 people (World Bank 2018), far from WHO recommended 1:600 (WHO 2007). AYUSH integration was introduced under the National Rural Health Mission, which mandated the co-location of a registered practitioner from one of the AYUSH systems alongside biomedical professionals in all public primary healthcare facilities. The policy documents do not provide an explicit definition of integration that clarifies policy processes or implementation. For example, the national integration guideline specifies ‘the outpatient services would be strengthened through posting/appointment on contract of AYUSH doctors over and with the [bio]Medical Officers’ (p. 34), separate AYUSH setup would be provided. (p. 34), and AYUSH drugs will be supplied (MoHFW 2005). In the health policy literature, TCAM integration refers to the incorporation of TCAM providers, practices, and products into mainstream biomedical health services and regulatory and financing structures (Nambiar et al. 2014). Elsewhere, the integration has been described as the ‘bringing together of different groups into unrestricted and equal association… blending, amalgamation and fusing’, while concurrently retaining their heterogeneity (Lakshmi et al. 2015, p. 3). This analysis considered integration aligning with the guidelines as the placement of AYUSH doctors within the mainstream primary healthcare system co-locating with biomedicine professionals, and associated phenomena of governance, to explore the role of the state in integration processes (MoHFW India 2005, Ministry of Health and Family Welfare 2017).

In the state where this study was conducted, AYUSH integration is governed in parallel by the Directorate of Health Services and Directorate of AYUSH—two directorial divisions within the Department of Health and Family Welfare. The AYUSH integration plan included developing a partnership relationship between Directorate of Health Services (responsible for medicine, supplies and wider governance) and Directorate of AYUSH (responsible for AYUSH doctors’ salary).

AYUSH integration aligns with India's ‘One Nation, One Health System’ vision aiming for unified healthcare by 2030 (Bhatt 2020). However, studies on AYUSH integration in India highlight a range of challenges, including the operationalization of medical pluralism (Khan 2006, Broom et al. 2009, Lambert 2012, Priya 2012, Patel et al. 2023), discrepancies in professional roles and practices (Josyula et al. 2016), ethical concerns, service delivery limitations, and remuneration issues (Lakshmi 2012, Mallick 2016). Power imbalances between professionals and inadequate governance further undermine policy implementation (Lakshmi 2012, Nambiar et al. 2014). While the AYUSH integration aims to institutionalize medical pluralism, evidence from both developed and developing countries suggest that TCAM integration often results in partial integration and increasing biomedicalization of TCAM (Anderson 1999, Mathpati et al. 2020). Biomedicalization is referred to as the concerted efforts to adapt the TCAM approaches of education and practices into the biomedical paradigm (Ning 2008).

Our prior empirical study highlighted the interconnectedness of administrative, facility-level, community, and societal contextual factors that influence AYUSH integration (Patel et al. 2023). The pre-existing administrative measures, resources, and capacity deficits limit access to AYUSH medicines and opportunities to develop relationships between biomedical and AYUSH doctors. Acceptance of AYUSH in rural communities, support from professional organizations and the media facilitate AYUSH integration by holding health systems accountable (Patel et al. 2023). In the context of Primary Health Centres (PHCs) we studied, AYUSH doctors work within a hierarchical structure dominated by biomedicine doctors (Patel et al. 2021). Biomedicine doctors use authoritative power to restrict resources, including funds and personnel, which undermine the AYUSH integration policy priorities. AYUSH doctors are directed by biomedical doctors to practice biomedicine, stepping beyond the boundaries of the AYUSH integration policy in a process of medical dominance we label ‘co-optation’ (Patel et al. 2021).

Despite growing scholarship on integration, the role of the state in actively or passively influencing AYUSH integration remains underexplored. Therefore, in this paper we draw from the same dataset to explore the role of the state in the AYUSH integration processes, and their dynamic implications for AYUSH doctors.

## Materials and methods

### Research sites

This study was conducted in an eastern Indian state recognized by the National Health Mission as a priority state due to poor health indicators. At the time of data collection, the state’s maternal mortality ratio was 119 per 100 000 live births, compared to the national ratio of 97 (SRS 2019). Three districts were selected based on their contrasting development indicators, health outcomes, geographical features, and availability of AYUSH colleges. We planned this purposive selection to collect distinct experiences among health system stakeholders. Within each district, data collection took place at one or more block-level Community Health Centre (CHCs) and/or at the smaller PHCs that sit under them. The selection of CHCs and PHCs was based upon the consent of the staff employed at those facilities to participate in observations and interviews. To protect participants’ privacy, all potentially identifiable details, including state, district, and site names have been replaced with pseudonyms, and any personal identifiers have been removed.

### Data collection and participants

To explore the range of factors influencing AYUSH integration and to incorporate both overlapping and contrasting perspectives, data were collected through qualitative interviews and non-participatory observations. An initial participant information statement was distributed across the selected CHCs and PHCs by district-level administrators. Prospective participants were recruited after they provided written consent, and all who consented were included in the study. Participants included AYUSH doctors, biomedical doctors, nurses, pharmacists, and district and state-level administrators. The lead author (G.P.) conducted all primary data collection and, although trained in pharmacy, did not occupy a clinical role in either biomedicine or any AYUSH system. G.P. is proficient in the local language, and interviews were primarily undertaken in this language, except for state-level health system administrators, who preferred to interact in English. During fieldwork, G.P. was identified as a PhD student, which meant that he held a student/learner, non-powerful, and non-state actor identity.

A total of 37 participants were interviewed to explore a range of perspectives (see Table 1). Interviews ranged from 20 to 70 minutes and were conducted using semi-structured interview guides. All interviews were conducted in the local language, audio-recorded (with participant consent), transcribed, and translated into English. The lead author worked closely with professional transcribers throughout the transcription process, reviewing the content to ensure accuracy.

**Table 1:** Characteristics of participants.

	Korhampur	Kolhaban	Soor	State-level
AYUSH doctors	5	7	3	0
Biomedical doctors	1	2	1	0
Nurses	0	2	1	0
Pharmacists	1	1	0	0
Health system administrators	2	4	3	4
Total (37)	9	16	8	4

In addition to interviews, over a 6-month period, the lead author conducted 19 days of observations in various PHCs and CHCs, observing and documenting activities such as patient consultations, staff interactions within health centres, facility-level meetings with community health workers, monthly district-level meetings with all staff, as well as community activities such as immunization and village health nutrition days. Preliminary findings from these observations informed subsequent interview discussions. This study received ethical approval from the Human Research Ethics Committee, the University of Newcastle, Australia (approval No. H-2017-0310).

### Data analysis

The themes noted in this paper, which focus on the role of the state in the AYUSH integration processes, represent a subset of themes identified in the broader study. An inductive coding frame was developed to capture important themes identified during data familiarization. We coded transcripts and observation notes using NVivo 12. The coded segments were read and re-read to identify organizing themes focusing on how the state played an active or passive role in AYUSH integration implementation and practices. To improve the credibility of the findings, a member-checking process was conducted among six participants. All participants were invited to review a summary of the themes. While 11 agreed to discuss these findings in detail, five withdrew due to other work commitments during G.P.'s time in the field. We used the notes from these member-checking discussions to refine and confirm the thematic conclusions.

Six key themes are outlined as subheadings in the results sections below, covering the role of different levels of government, issues with nomenclature, and the drivers of biomedicalization.

## Results

### Role of the government as the employer

India’s public-sector health workforce is stratified into permanent and contractual appointments, a structure that classifies and governs job security, pay, and career mobility. AYUSH doctors, although officially designated ‘AYUSH medical officers’ in PHCs, are almost always employed on rolling short-term contracts, unlike most biomedical professionals. As a result, AYUSH doctors face risks of dismissal, lower wages, no pathways to postgraduate-level apprenticeship, limited career development and limited transfer or promotion opportunities.

A biomedical doctor normalized the differences and framed the gap as a state decision:

Yes, they have a lesser salary, but it’s not too bad. It is the government’s decision. They have joined this system recently, so maybe the government is assessing them. (Kolhaban_Biomedical doctor_03)

AYUSH doctors themselves perceive the design as exclusionary:

The government gave us a lower position when they gave us the contractual status. I feel trapped. I am the same person who I was eight years ago… And, we are like, once we join here, I may die in the same position. (Kohrampur_Homeopathy_02)

The discrepancy in pay and employment security ultimately aligns with a pre-existing institutional hierarchy that shapes workplace dynamics: those with permanent contracts enjoy greater respect, prestige, and inter-professional authority, while contractually employed AYUSH doctors frequently encounter marginalization. As a result, these inequities affect how biomedical professionals perceive and interact with AYUSH doctors, reinforcing hierarchical divisions within the integrative health settings.

Now it is 12:30[PM], and nobody is here [in the PHC], despite this being official duty time. If the MO [biomedical doctor] had been here, no one would leave before the MO leaves the office; it does not matter whether it is 1 or 5 pm. (Kohrampur_Ayurveda_01)

Later in the interview the same participant adds:

There are differences in respect between them [biomedical professionals] and us. They don’t bother to inform or even talk to us. Like, right now I don’t know who is in the hospital. If something happens then, I don’t know whom to ask for help. (Kohrampur_Ayurveda_01)

These inequities extend into social life. Insecure, modest incomes limit AYUSH doctors’ access to quality housing, education, and healthcare, especially in rural postings, establishing their subordinate status both within the profession and the wider community.

### Inter-governmental dissociation

At the state level, inter-governmental collaboration in AYUSH integration is intended to establish interactions between government agencies, the Directorate of Health Services and the Directorate of AYUSH—both responsible for integrative healthcare services. However, these collaborations often lack shared principles, governance structures, and expectations. Limited communication and coordination between the two directorates hinders the resolution of AYUSH doctors’ concerns regarding their required medicines—which were often in short supply—and logistics, which further creates confusion over accountability and limits the integration process. Discussing the problem of the lack of Ayurveda medical supplies, an Ayurveda doctor stated:

I submit my application to the MOIC [biomedical doctor and administrator] for medicines, if it cannot be resolved locally at the CHC level, then I don’t know what happens [at the state level]. There is no [other] way to submit a requisition to the Directorate of AYUSH. (Soor_Ayurveda_07)

Contrastingly, at the local level, collaborative initiatives occur outside the health system. For instance, the Public Works Department (responsible for public infrastructure) worked with AYUSH doctors to improve road infrastructure and access to primary healthcare centres. Through this collaborative action, the roads leading to health centres were repaired, which facilitated access to PHCs:

I was worried about the road… I just wrote a letter to the Block Development Officer about the [road] situation… the PWD people got involved within 2–3 weeks, and the road work began. That was helpful for everybody to reach the hospital easily. (Kolhaban_Ayurveda_06)

While such inter-sectoral efforts with non-health departments were productive, governance, and collaboration between the health organizations remained relatively fragile. A state-level administrator expressed that collaborations are an ongoing learning process and said:

In the beginning [of integration], everything was done by the National Health Mission. After 2014 [and] the formation of the Directorate of AYUSH, we shared the responsibilities. Even if we do our respective work according to the guidelines, then also we should not assume that everything will be fine. You can’t expect 100% success, but everybody needs to learn from the mistakes and try to work as a team. (State_Administrator_10)

### Biomedicalization of Ayurveda, Yoga and Naturopathy, Unani, Siddha, and Homeopathy integration

Despite recognition of medical pluralism as a goal in AYUSH integration policies, the Directorate of Health Services treats it as secondary. This marginalization occurs due to a lack of clear timelines or measurable objectives, unlike other national health programmes with defined targets. As a state-level administrator explained:

Programmes like immunisation have explicit timelines and consequences for non-achievement. But we don’t have any timeline for medical pluralism, how would someone measure pluralism in a year or six months? Hence, we do not prioritise and are not accountable for pluralism, but in programmes, we are. (State_Administrator_13)

These issues appear to be entrenched in the governance of the Directorate of Health Services, where the AYUSH doctors are utilized to achieve health system goals associated with biomedicine practices. The state administration has developed training initiatives, such as the skilled birth attendant programme, expanding AYUSH doctors’ biomedical skills to meet health service goals. Thus, AYUSH doctors are often allocated biomedical tasks, resulting in widespread task-shifting from their AYUSH systems towards biomedicine practices.

At facility level, AYUSH practitioners face practical implications due to task shifting. One Ayurveda doctor noted: In the beginning [7–8 years back], I had [AYUSH] medicines, I was happy and the patients too. But these days, I either have to tell patients to buy from outside, prescribe biomedicine or refer them to the other [biomedicine] OPD. Now I am a referral person only. What to do? (Soor_Ayurveda_07)

Training AYUSH doctors in biomedicine and assigning them biomedically orientated tasks has expanded their responsibilities beyond their respective systems of medicine. While some AYUSH doctors perceive biomedicalization as compromising their professional authenticity, others appreciate biomedical diagnostics and procedures for improving patient trust and credibility in their consultation. For example, one Ayurveda doctor said:

Patients feel happy. When I use a stethoscope and fluorescent light to check their tongue and eyes, they feel much comfortable about their diagnosis. Even the tests are useful. The patients also get more satisfaction that they were being treated carefully after a comprehensive check and discussions. (Soor_Ayurveda_10)

To mitigate biomedicalization, several participants recommended transferring governance of AYUSH entirely to the Directorate of AYUSH. While maintaining shared physical integrative facilities, they argued that professional oversight, salary management, and medicine procurement should be independent of biomedical administration. This autonomy, they suggest, would facilitate genuine AYUSH integration and better recognition of their professional practices.

If the Directorate of AYUSH will control everything, they understand our medicine, our skills and specialities. So, whatever happens, we will inform the AYUSH director in [state capital]. This is the way for actual AYUSH integration. (Kolhaban_Homeopathy_10)

Nonetheless, incorporating certain biomedical practices remains beneficial in improving patient satisfaction and enhancing AYUSH practitioners’ professional status within integrative healthcare settings.

### Role of local government

Local governance bodies, such as the ‘Gaon Kalyan Samiti’ (VHSNC) and the Sarpanch (the elected official in the Panchayati Raj system), sometimes contribute to the management of PHCs by supporting AYUSH doctors. Their support is highly context-specific, responding to the particular needs of remote primary healthcare facilities that face chronic shortages of biomedicine staff and medicines. In some instances, Panchayat leaders have channelled local funds to purchase AYUSH supplies:

In a Panchayat meeting…I spoke about the [medicine] problems and how people don’t get appropriate Ayurveda medicines… Then, some days later, 4–5 people came here [PHC], and they gave me 9000 rupees to buy essential medicines. I was shocked and happy to see people have much trust in me. They are not wealthy; they have their problem with money. (Soor_Ayurveda_08)

Thus, decentralization facilitates community awareness of PHC services through local governance, community participation and direct engagement with AYUSH integration. Several AYUSH doctors described instances of villagers collectively raising funds to procure medicines, while local governance committees help manage crowds during outreach camps, coordinate schedules and disseminate information on national health programme activities.

This collaboration between local agencies and AYUSH doctors functions as a social contract: community groups contribute financial and logistical resources; AYUSH doctors provide labour and expertise. Yet, this form of social contract remains tenuous. Local government organizations have no official mandate in the AYUSH integration structure, their participation remains voluntary and inconsistent across different regions. Further, AYUSH doctors recognize that local agencies are unlikely to offer financial support indefinitely, highlighting the fragility and variability of this grassroots-driven assistance.

### Ayurveda, Yoga and Naturopathy, Unani, Siddha, and Homeopathy medical officer: issues with the nomenclature

The state standardized and grouped together practitioners from diverse AYUSH systems under the unified titles of ‘AYUSH doctors’ or ‘AYUSH Medical Officers’ as part of their integration into primary healthcare. In practice the undifferentiated title blurs professional identities, confuses patients and constrains therapeutic choice. An Ayurveda doctor explained:

How would they[patients] know if I am Ayurveda or Homeopathy doctor? Sometimes patients ask… So, in cases of children, the parents prefer Homeopathy, and if I write the Ayurveda medicines, they ask for the small-small tablets [Homeopathy]. That’s uncomfortable and waste of my and patient’s time. (Soor_Ayurveda_08)

A homeopathy doctor echoed the problem:

It is not that easy. There are six-seven systems of medicines, which is almost everything. How would a patient know who is who? (Kolhaban_Homeopathy_09)

Even a state-level senior administrator acknowledged the ambiguity in terminology and asked rhetorically whether ‘one doctor equals five’:

I went to Vidal [pseudonym] dispensary; want ayurvedic medicines, but there I found an “AYUSH doctor”. When I asked about his stream & he replied Homeopathic. It means…I won’t get the medicine I want…if we say AYUSH doctor, what it means, one doctor is equal to five? That needs to be addressed. (State_Administrator_10)

Clearer designation based on systems of medicine matters because modalities contribute differently to service delivery. At the facility level, it was observed that Ayurveda doctors tend to emphasize dietetics and medicinal plants, advising turmeric and ‘Gudmar’ (Gymnema) during community-level non-communicable disease sessions, whereas homoeopathy doctors focus more on physiological activities of the body. Nevertheless, their day-to-day work increasingly converges. National programmes impose uniform objectives, timelines and reporting formats, obliging every practitioner (whether Ayurveda or Homeopathy), to implement immunization programmes, antenatal check-ups and health education camps according to biomedical-based protocols. Thus, the integration policy concurrently minimizes philosophical distinctions and subsumes AYUSH under broader public health targets.

Despite the complexities involved in the terminology, it was found that the AYUSH doctors working in the primary healthcare settings manage to work together while sharing the same restrictive integration policy. Although a practitioner from only one AYUSH system of medicine is employed in each primary healthcare centre, the group of AYUSH doctors working in the same regions meet at various health system events, such as CHC-level monthly meetings and district-level meetings and training. For example, one such event was observed and recorded in the fieldnotes:

In Kohrampur, 8–10 AYUSH doctors attended the meeting…. organised by the Chief District Medical Officer (CDMO) to share work plan for health and wellness centres. The AYUSH doctors…gathered to discuss their role, travel reimbursement and maintaining documentation of expenses. (Kohrampur_OBS_09)

Such gatherings build interpersonal trust among AYUSH doctors across systems of medicine and reveal shared working conditions—contractual status, modest pay, and limited stocks of system-specific medicines. However, the community of AYUSH doctors still has little to no voice in the district-level decision-making.

The integration challenges and vulnerable position of the AYUSH doctors have been discussed in earlier sections. Despite the challenges, the AYUSH doctors could not leave their jobs and continue to practice while being involved in the integration, because the market demand for AYUSH practices is limited.

### The role of the government in shaping the market and availability of health services

The opportunities and structure of the market availability for healthcare practices, other than at the government facilities, are shaped by the creation of institutions with different reputations, funding and quality by the government. The Indian government regulates the education, training and legitimate spheres of the AYUSH practices. The state also has an indirect role in creating the market for AYUSH practices. The higher funding from the government for the mainstream biomedicine, compared to the AYUSH systems, creates better infrastructure and institutional rankings, which leads to an overarching perception that biomedicine treatments are better than AYUSH treatments, along with having greater prestige and institutional status, as one health system administrator explained:

Allopathy is getting maximum money from the system for the last maybe 40–50 years, so there are better hospitals, better technology, and better medical schools. So, people think they are better than AYUSH. So that’s the reason even if some patients go to the private hospital, they spend their money at allopathy private clinics. (Soor_Administrator_09)

The employment market availability for the AYUSH doctors is less than for the biomedical doctors. An Ayurveda doctor explained why there are few employers for AYUSH doctors other than the government:

If I leave the job because of the problems here, where do I go to work? Either here, or I have to start my own clinic, which is risky… For them [biomedicine], there are a lot of private hospitals like Apollo, Max, but we don’t have that option. (Kohrampur_Ayurveda_02)

Elsewhere, a health system administrator also suggested:

If the government invests more in AYUSH, people will increasingly visit AYUSH health centres. That will catch the eyes of private parties to come-up with their hospitals. That will create more jobs and opportunities for AYUSH doctors. (Soor_Administrator_07)

At the time of data collection, the government has no plans for creating alternative private labour market opportunities for AYUSH doctors. This latent role of the government is linked with the vulnerable position of the AYUSH doctors in the integrative primary healthcare settings, as they were living in a situation of employment or unemployment, depending on being recruited or dismissed by the government. The study participants further suggested that the integration-related organizational changes and the professional development of the AYUSH doctors can both occur with heterogeneous market forces, where different employer organizations compete with each other to meet the demands for services in the market. For example, an Ayurveda doctor said:

The Ayusman Bharat [government’s free health insurance for poor people] was implemented in 2016*-*17. Why didn’t they include AYUSH in that insurance? It is only about allopathy. If they had included, that could have encouraged AYUSH practitioners to broaden their infrastructure and services delivery because they would have received patients with that insurance. (Kohrampur_Ayurveda_01)

The government has played a crucial role in the unavailability or limited availability of a market for AYUSH doctors beyond the integration programme at the PHC level. A Homeopathy doctor also recommended that there should be AYUSH promotions in the public health system:

If you go to all the PHCs and CHCs in the entire state, you will not find a single signboard for the AYUSH. But for every health programme [immunisation, Janani Surakhya Yojana [Mother Protection Scheme], Leprosy, Tuberculosis control etc]… there are big hoardings, and sometimes even television advertisement to inform people about the programmes to create awareness for people to avail health services. Why can’t they do the same for AYUSH? At least there should be some posters, hoarding in the villages and towns. (Kohramour_Homeopathy_05)

This quote suggested that there is a need to promote AYUSH services and create awareness among people about the available AYUSH services. These sorts of government promotions can create a market for the AYUSH doctors by strengthening public trust and awareness about the AYUSH services in the integrative settings.

## Discussion

**Figure 1:**
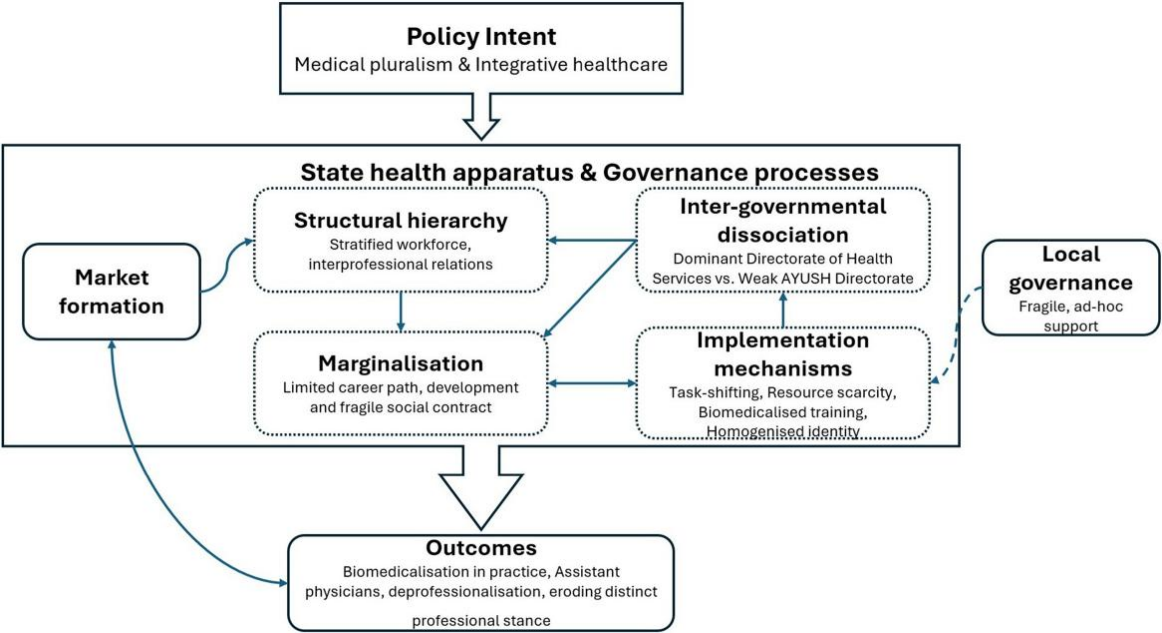
Thematic framework of the role of the state in AYUSH integration.

AYUSH systems and their integration have been national health policy priorities, with government commitment demonstrated through continuing support for AYUSH integration and its reiteration in the Ministry of Health and Family Welfare 2017 and National Education Policy 2020. However, the embedded biomedicalization, medical dominance and health system structures orientated around biomedicine were not addressed in implementation development. Therefore, AYUSH integration has been organized without commensuration structural adaptation, leaving its implementation to unfold in an *ad hoc* approach within existing mainstream governance arrangements—a recurring characteristic of top-down health policy reforms (Fig. 1).

Poor organizational communication and fragmented accountabilities in the government were significant barriers to inter-governmental collaborations, representing what (Anaf et al. 2014) described as unsuccessful ‘partnership relationship’. The role of the government in strengthening the integration processes could have been improved through greater professional autonomy and improved communication between two directorate heads, followed by demonstrated leadership to raise awareness amongst the state-level officials. This type of initiative could help advance the government’s intention to bring medical pluralism. However, in practice, the form and function of the AYUSH integration shifted from policy-level vision of medical pluralism, to one of co-optation at the primary healthcare level (Patel et al. 2021).

Despite these limitations, the integration process still showed the health systems function as complex adaptive systems in which collaborations between government departments existed (Barthwal and Sah 2008). In such systems, local collaborative initiatives sometimes mitigate problems with health facilities. This is evidence of how small-scale, decentralized efforts can influence the broader system in the absence of strong institutional coordination. However, without unified leadership or clear strategies, these grassroots efforts remain scattered. Strengthening inter-governmental collaboration and accountability is important for moving beyond isolated successes.

The AYUSH integration activities, such as the biomedical-based training of AYUSH doctors and their adoption of biomedical tools during consultations, exemplify what (Ning 2008) conceptualized as ‘biomedicalization’—a deliberate attempt of professionals and systems to gain legitimacy by transitioning a TCAM model of practices to a biomedical paradigm in biomedicine-based facilities. These activities highlighted a process of deprofessionalization, redefining AYUSH doctors as assistant physicians through persistent co-optation (see Patel et al. 2021). Therefore, the integration policies and current practices have contributed to a shift from medical pluralism in policy to co-optation in practice, restructuring AYUSH doctors’ roles to fit a biomedical mould. This shift also conceptually aligns with biomedicalization, whereby AYUSH doctors, working under the policy directives, gradually internalize and reproduce biomedical approaches as part of their regular clinical activities. Shortages of Ayurveda or Homeopathy medicines, for instance, were rarely seen as state failure but as AYUSH doctors-level problems resolved through biomedical practices of AYUSH doctors. Over time, these pathways encouraged AYUSH doctors to adapt by learning biomedical procedures or by referring patients to biomedical doctors. Thus, governance of AYUSH doctors in the integration policy processes operated less through overt coercion and more through policies, expectations, and administrative structures that normalize a biomedical structure of AYUSH integration. Cross-learning through AYUSH training for biomedical doctors is stated in the National Education Policy 2020, which recommends reforms to include fundamentals of AYUSH in biomedical curricula (Ministry of Human Resource Development 2020, p. 50). However, at the time of data collection, these reforms had not yet been implemented.

The study findings are aligned with the concept of policy networks and ‘institutions, interests, and idea-based’ theories of policy change (Shearer et al. 2016). The national and state government institutions with their interests and goals were significant in (re)shaping AYUSH integration approaches and influencing prospects of AYUSH practices and success. This study contributes to knowledge by identifying the changes in the policy processes over time while they were being translated into an implementation plan and the demonstration that the changes were typically implemented by the most influential and dominant institutional agencies with different sets of institutional interests. For example, the Directorate of Health Services was the dominant department that prioritized the activities of the health system that were commensurate with their programme implementation plan.

While policy documents promote ‘AYUSH’ as a unified category, in reality it involves multiple systems of medicines, each with its own philosophical principles and practices. By consolidating them under a single label, the state effectively imposes a monolithic identity that masks their distinctiveness. This homogenization aligns with broader patterns of state-driven medical dominance, where pluralistic TCAM are subsumed under standardized regulatory frameworks to facilitate governance, eroding their particular epistemologies and practices (Ashworth and Cloatre 2022).

Systemic shortfalls in the state’s health system structure and implementation processes enabled PHC and district-level decision-makers to exert authority to shape AYUSH integration practices. Although policies endorse medical pluralism, state apparatus, and agencies influenced whether (and how) AYUSH doctors could apply their skills in PHC contexts. AYUSH doctors were assigned to conduct community immunization programmes or other national health prevention and promotion activities, limiting opportunities to practice their own systems. Despite formal guidelines, frontline decision-makers prioritized biomedical programmes with targets. As these micro-level choices accumulated, they overshadowed the broader medical pluralism objective, effectively repositioning AYUSH doctors to fill biomedical workforce needs.

Another significant finding of this study is built on the concept of complexity theory in the policy evaluation (Haynes 2008), where the biomedicine-based mainstream health system’s reductionist approach for monitoring and policy evaluation does not capture subjective value-based aspects of practice. For example, the primary healthcare activities that could be numerically measured, such as the number of patients screened or the number of institutional deliveries conducted, were prioritized by the state and local administrators, marginalizing the activities that could not be measured numerically, i.e. medical pluralism. These findings align with critiques highlighting that public policy evaluations grounded exclusively in a ‘restricted positivist framework’ (Byrne 1998, Barnes et al. 2003, Carson and Kerr 2017) often undermine experiential and societal outcomes. Therefore, we recommend incorporating more subjective values and measures into AYUSH integration’s performance monitoring.

The inequities were especially evident in employment patterns. AYUSH doctors work under precarious contracts and have limited opportunities outside the public sector. State insurance schemes (like Ayushman Bharat) generally exclude AYUSH treatments, discouraging prospective private investors from establishing AYUSH clinics or hospitals. As a result, AYUSH systems of medicine and their practitioners remain trapped in a cycle of low demand, inadequate infrastructure, limited employment opportunities and professional development. This has created a system where AYUSH doctors either remain reliant on unstable contractual government contracts or gravitate towards biomedical tasks to ensure steady employment.

## Limitations

This study uses the term AYUSH, although only Ayurveda and Homeopathy doctors participated in the research. Perspectives of other AYUSH doctors (such as Unani, Siddha, and Yoga and Naturopathy) are not represented and may differ. However, given that all AYUSH doctors are governed by the same national policy, their experiences are likely to be similar.

The study does not incorporate patients’ perspectives on AYUSH integration, which may overlook patient-specific challenges and the state’s role in shaping public perceptions of AYUSH. Future research should include patient perspectives for a more comprehensive understanding of AYUSH integration.

Health policies are dynamic and subject to change. Changes in policy guidance and practices may lead to different challenges or potentially address some of those identified in this paper.

We did not explore philosophical implications of grouping diverse AYUSH systems together, nor how this aggregation shapes their philosophical practices or broader discourses. Future research should explore how the distinct epistemic strengths of each system are implemented in practice.

## Conclusion

Integration policy implementations were shaped by a combination of feedback loops, resource scarcities, and institutional partnership. These created a system that responded to practical constraints rather than to the normative vision of medical pluralism. The AYUSH integration faced complex issues that had their origins in the government administration and its integration policies, which were sometimes beyond the scope of the primary healthcare system. The ongoing integration affected individual AYUSH doctors through the redistributive goals of the health system created by the state. However, the AYUSH doctors involved in the integration were also susceptible and vulnerable to the broader professional, economic and social developments. The state, as a healthcare institution, did not exist only in the guidelines and policy briefs but rather was evident in the day-to-day processes of AYUSH integration and the interactions between the health system actors and the healthcare facilities. The combined actions of the government and its agencies implementing the integration had a significant role in changing the integrational pathways and biomedicalization of AYUSH integration. At the micro-level, local government influence was neither monolithic across the local contexts nor consistent due to the absence of guidelines and direct accountability. Strengthening formal links between decentralized local governance and health-system structures would therefore be essential to sustain community-level gains and improve AYUSH services in underserved areas. The integration policy and related documents must clearly delineate the definition of integration, its characteristics and procedures, the implementation mechanisms, and the outcome measures for assessing the policy's effectiveness and its implications on medical pluralism.

This study identified how structural and procedural complexities within the state’s policy apparatus actively produced conditions that narrowed AYUSH practice. This study highlights how state agencies redirect policy intent and contributes to the understanding of the state’s constitutive role in policy implementation. These insights extend beyond AYUSH integration and contribute to health policy discourses to analyse how structural frictions and the state shape the trajectories of complex reforms.

## Author contributors

Conception or design of the work: G.P., C.B., and A.T. Data collection: G.P. Data analysis and interpretation: G.P., C.B., and A.T. Drafting the article: G.P. Critical revision of the article: G.P., C.B., and A.T. Final approval of the version to be submitted: G.P., C.B., and A.T.

## Data Availability

The data underlying this article cannot be shared publicly due to participant privacy and confidentiality. Participants provided consent on the condition that their data would not be shared publicly. Fully anonymised data may be made available on reasonable request to the corresponding author.

## Reflexivity statement

The authorship team is comprised of one man and two women. In accordance with principles of decolonizing global health, this study was led by the local researcher (G.P.), who was born and raised in the state where the study was conducted. G.P. led all aspects of the research, and the international supervisors—co-authors in this paper (C.B. and A.T.)—intellectually contributed to the research and this paper. Although G.P. is trained in pharmacy, he did not occupy a clinical role in either biomedicine or any AYUSH system during the study. Interviews were primarily undertaken in the local language, in which G.P. is proficient; English was utilized only for state-level health system administrators who preferred that medium. During fieldwork, G.P.’s positionality was framed as a ‘student/learner’. This non-powerful, non-state actor identity helped mitigate power imbalances with participants. This studentship status was transparently communicated in the Participant Information Sheet and the state government approval letter obtained prior to data collection and was distributed to all district-level health system administrators.
